# H-NS, Its Family Members and Their Regulation of Virulence Genes in *Shigella* Species

**DOI:** 10.3390/genes7120112

**Published:** 2016-12-01

**Authors:** Michael A. Picker, Helen J. Wing

**Affiliations:** School of Life Sciences, University of Nevada Las Vegas, Las Vegas, NV 89154-4004, USA; pickerm3@unlv.nevada.edu

**Keywords:** H-NS, *Shigella*, transcription, xenogeneic silencing, virulence gene expression, silencing mechanisms, anti-silencing mechanisms

## Abstract

The histone-like nucleoid structuring protein (H-NS) has played a key role in shaping the evolution of *Shigella* spp., and provides the backdrop to the regulatory cascade that controls virulence by silencing many genes found on the large virulence plasmid. H-NS and its paralogue StpA are present in all four *Shigella* spp., but a second H-NS paralogue, Sfh, is found in the *Shigella flexneri* type strain 2457T, which is routinely used in studies of *Shigella* pathogenesis. While StpA and Sfh have been proposed to serve as “molecular backups” for H-NS, the apparent redundancy of these proteins is questioned by in vitro studies and work done in *Escherichia coli*. In this review, we describe the current understanding of the regulatory activities of the H-NS family members, the challenges associated with studying these proteins and their role in the regulation of virulence genes in *Shigella*.

## 1. Introduction

*Shigella* spp. are gram-negative, intracellular bacterial pathogens in the γ-proteobacteria that cause bacillary dysentery in humans. Four *Shigella* species have been described: *S. dysenteriae, S. flexneri, S. boydii* and *S. sonnei*. Each of these species is primarily transmitted by the fecal–oral route and invades cells of the colonic epithelium. The destruction of the colonic epithelial barrier and the severe inflammation that follows causes the symptoms of this disease (shigellosis, also known as Marlow syndrome). *Shigella* spp. are estimated to cause more than 88 million cases of shigellosis each year [1], and more than 40,000 deaths worldwide [2]. These statistics, the rise of antibiotic resistant strains and the absence of approved vaccines underscore the need for an improved understanding of the molecular biology of this pathogen so that novel antibacterial strategies can be found and implemented.

*Shigella* spp. are close phylogenetic relatives of *Escherichia coli*. All four *Shigella* species harbor a large virulence plasmid (~180–220 kb) [3], which is essential for their pathogenicity. Genes encoded by the virulence plasmid are necessary for host cell invasion, intra- and intercellular spread and host cell manipulation via the type three secretion system [4]. Evidence suggests that the acquisition and maintenance of the virulence plasmid, its subsequent evolution and the present day transcriptional regulation of these virulence genes is tied to the chromosomally-encoded histone-like nucleoid structuring protein, H-NS [5,6,7].

As a nucleoid structuring protein, H-NS organizes and compacts DNA, but it also globally regulates the expression of ~5% of all genes in *E. coli*, many of which are involved in the transcription, translation and the production of cell envelope components needed for adaptation to varying environments [8]. H-NS preferentially binds AT-rich DNA, rendering it transcriptionally inactive or silent [9,10]. The silencing of the horizontally acquired AT-rich DNA has been termed xenogeneic silencing [9]. In *Shigella*, this H-NS-dependent process is thought to have allowed the initial acquisition and long-term maintenance of the large AT-rich (up to 70% in many ORFs) virulence plasmid without the newly acquired genes compromising the organism′s fitness. The eventual integration of these genes into existing transcriptional networks is also predicted to have been aided by H-NS-mediated silencing (reviewed in [7,10,11,12]). Today, the ecological fitness of *Shigella* spp. both ex vivo and in vivo is tightly connected to the silencing of virulence genes by H-NS. Outside of the host, transcriptional silencing avoids the costly production of proteins that provide no benefit to *Shigella* in the external environment [6]. In contrast, within the host, silencing mediated by H-NS provides the backdrop for the precise and hierarchical expression of virulence genes that occurs in response to environmental cues and signals encountered within the host environment [5,13]. Consequently, H-NS has had a profound effect on the evolution of *Shigella* spp. and continues to play a central role in the regulation of virulence genes in this group of important human pathogens.

In addition to H-NS, *Shigella* spp. contain up to two H-NS paralogues. The first paralogue, StpA, is chromosomally encoded and found in all *Shigella* spp. [14]. In contrast, the second paralogue, Sfh, is carried by an R27-like plasmid that is found exclusively in *S*. *flexneri* type strain, 2457T [15]—a clinical isolate that is commonly used in studies that focus on the molecular basis of an infection. Both StpA and Sfh have been proposed to serve as “molecular backups” for H-NS because these proteins can transcriptionally silence virulence genes in *Shigella* in mutants that lack *hns*. However, different nucleic acid binding activities and expression profiles of these proteins (described in more detail later; [15,16,17]) raise questions about the apparent redundancy of StpA and Sfh, their respective and combined activities and their interplay with other transcriptional regulators of the *Shigella* virulence gene cascade. Here, we review our current understanding of H-NS, its two paralogues and their role in the regulation of virulence genes in *Shigella* species.

## 2. H-NS and Its Role in the Regulatory Cascade Controlling the Transcription of Virulence Genes in *Shigella*

### 2.1. The H-NS Protein and Its Interactions with DNA

**Figure 1:**
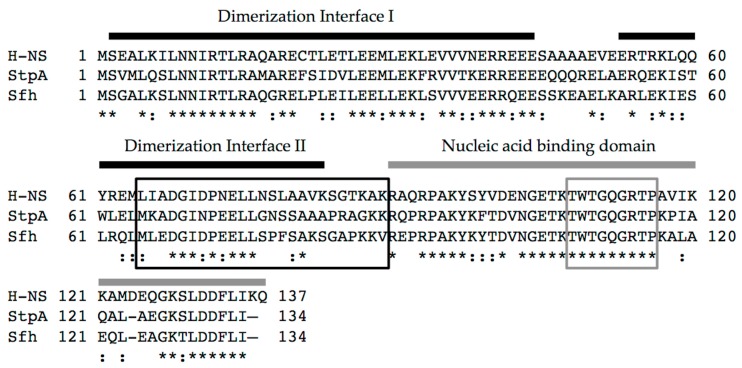
Alignment of the primary protein sequence of histone-like nucleoid structuring protein (H-NS), StpA, and Sfh from *S*. *flexneri* strain 2457T. Functional domains of H-NS are indicated. Two thick black bars represent discrete interfaces involved in dimerization that are needed for the formation of higher order oligomers [22]. The thick grey bar indicates the nucleic acid binding domain. Boxed regions represent either the flexible linker region (black) or the DNA binding motif (grey) [23]. Conserved residues are indicated by asterisks, and similar residues are indicated by colons. Percent amino acid identity: H-NS and StpA (56.2%), H-NS and Sfh (59.1%), StpA and Sfh (61.9%).

The H-NS protein is encoded by a gene located in the *ter* macrodomain of the chromosome of both *E. coli* and *Shigella* spp. In these two closely related organisms, H-NS is 100% identical, making in vitro studies on the *E. coli* H-NS protein directly applicable to the *Shigella* protein. H-NS is small (15.4 kDa), highly abundant (20,000 copies per cell in stationary phase cultures [18]) and functions as a dimer or as a larger multimer [19]. Each protein monomer is comprised of two structural domains separated by a flexible linker: the N-terminal domain is directly responsible for dimerization/oligomerization, and the C-terminal domain confers DNA binding activity ([20,21,22]; Figure 1).

The ability of H-NS to transcriptionally silence genes can be explained by its DNA binding preference and the nucleoprotein complexes that form. High-affinity binding sites for H-NS have been found in *E. coli* [24,25], leading to a proposed consensus binding site (5′-AATTTATCGA-3′; [25]). More recently however, the width of the DNA minor groove has been demonstrated to primarily govern the DNA binding preference of H-NS, with H-NS preferentially binding to DNA with narrow minor groove widths [26]. Interestingly, an ATATAT motif [26], which is present in the two identical high affinity binding sites found in the *E. coli proU* operon, 5′ AATATATCGA 3′ [24,25], is predicted to narrow the minor groove to 3.5 Å (compared to 5.7 Å expected in B-DNA; [27], explaining why these high-affinity sites bind H-NS so well [24].

Once bound to a high-affinity region, H-NS oligomerizes along DNA into regions with lower affinity [25,28], leading to the formation of large H-NS:DNA complexes. Two H-NS nucleoprotein structures have been visualized and studied using atomic force microscopy [29,30] and single molecule experiments [21,31]. H-NS bridges form when two discrete DNA regions are brought together by H-NS. In contrast, nucleoprotein filaments form when H-NS oligomers coat long, contiguous stretches of DNA. Both of these H-NS:DNA structural complexes are predicted to be involved in the silencing of virulence genes in *Shigella* [32,33,34].

### 2.2. The Role of H-NS in Shigella Virulence Gene Regulation

**Figure 2:**
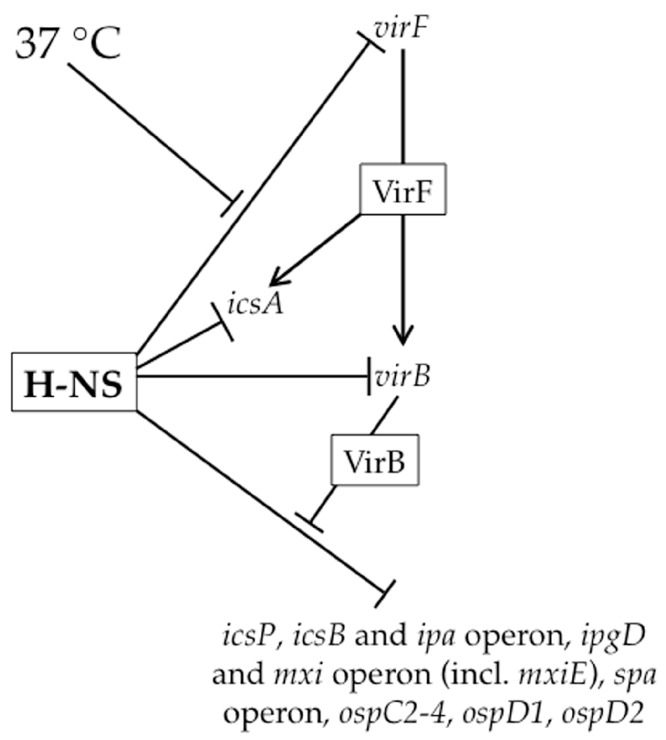
Overview of the regulatory virulence cascade in *Shigella* spp. H-NS silences virulence genes at each step of the virulence cascade. H-NS-mediated silencing of the *virF* promoter is alleviated upon a switch to 37 °C. VirF subsequently directly activates the *icsA* promoter as well as the *virB* promoter. Next, many genes on the virulence plasmid are upregulated by VirB via a counter-silencing mechanism.

Under non-physiological conditions (osmolarities lower than physiological, pH below 7.4 and temperatures below 37 °C), H-NS silences many genes encoded by the *Shigella* virulence plasmid, including those encoding the transcriptional regulators of the virulence gene cascade ([35,36,37,38,39]; Figure 2). Upon a switch to 37 °C, a temperature-dependent remodeling of the H-NS:DNA complex located within the *virF* promoter region triggers the production of VirF, the master regulator of this regulatory cascade ([32,40]; Figure 2). Consequently, the thermally-induced modulation of a H-NS:DNA complex can be considered the key event that triggers the regulatory cascade controlling *Shigella* virulence.

**Figure 3:**
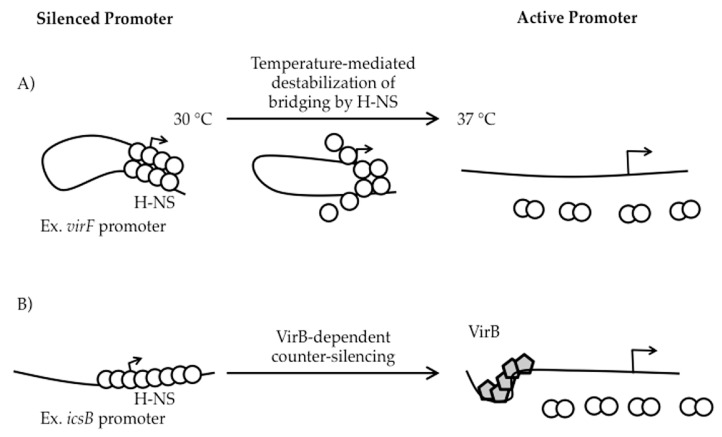
Temperature- and protein-dependent mechanisms of counter-silencing in *Shigella.* (**A**) At 30 °C, the *virF* promoter is silenced through H-NS-mediated bridging of two discrete binding regions separated by a region of DNA curvature. As the temperature increases to 37 °C, movement of the curvature remodels the H-NS bridging complex leading to *virF* expression [40,55]; (**B**) H-NS directly silences the *icsB* promoter. VirB binds DNA, induces a local DNA bend and oligomerizes along the DNA, which destabilizes the downstream H-NS silencing complex [56].

At the *virF* promoter, two discrete H-NS binding regions, centered at -250 and -1 relative to the *virF* transcription start site, flank a temperature-dependent DNA “hinge” region [32,40,41]. At temperatures below 32 °C, the relative bending and rotational orientation of the two H-NS binding regions are optimized for H-NS bridging ([40]; Figure 3A). Since one of the H-NS binding regions overlaps the core promoter elements, the formation of the H-NS bridging complex is thought to occlude RNA polymerase (RNAP) from the *virF* promoter [40]. As the temperature increases to 37 °C, movement of the curvature center disrupts the H-NS-silencing complex, allowing the promoter to become accessible and *virF* transcription to proceed ([40,42]; Figure 3A). These regulatory events showcase how temperature can induce changes in DNA topology and cause H-NS bridging structures to be remodeled, leading to the relief of H-NS-mediated silencing.

Once VirF, an AraC family member, reaches threshold levels, it up-regulates the transcription of *icsA* [43] and *virB* ([33]; Figure 2). The *icsA* gene encodes the determinant for *Shigella* actin-based motility [44,45], whereas *virB* encodes the next transcriptional regulator of the regulatory cascade [33,46]. While H-NS appears to silence transcription of both of these genes ([33,39,47]; Figure 2), we focus on the regulation of *virB* because of its integral role in the virulence gene cascade. At the *virB* promoter, only a single H-NS binding region has been identified that overlaps the core promoter elements [33], suggesting that transcription of *virB* is silenced by an H-NS nucleoprotein filament that occludes RNAP from this promoter. Indeed, H-NS-dependent silencing of *virB* can be observed using in vitro transcription assays. While VirF increases *virB* transcription in these in vitro assays, it does so only in the absence of H-NS [33]. Consequently, the authors conclude that VirF does not function to counter H-NS-mediated repression, but rather serves as an essential transcriptional activator of the *virB* gene once H-NS-mediated repression has been relieved [33]. To date, the event that relieves H-NS-mediated silencing of the *virB* gene remains unclear.

The production of VirB is a critical step in the *Shigella* virulence gene cascade because it relieves H-NS-mediated silencing of virulence genes that has not been alleviated by temperature alone. VirB up-regulates greater than thirty virulence plasmid genes [48,49,50,51,52,53,54], including those found within the 30 kb *ipa-mxi-spa* region, which encode structural components of the type III secretion system and their effectors, the MxiE transcriptional regulator (Figure 2) as well as several other genes outside of this region. The activity of VirB as a transcriptional anti-silencing protein is demonstrated by work at the *icsB*, *icsP* and *ospZ* promoters, where VirB has no effect on promoter activity in the absence of functional H-NS [34,50,54]. If VirB solely functions to relieve transcriptional silencing by H-NS, as predicted by the experiments described above, then any gene found to be directly regulated by VirB will be silenced by H-NS.

Indeed, all three VirB-regulated promoters found within the *ipa-mxi-spa* region are silenced by H-NS at 30 °C [57], but the silencing and anti-silencing of the two divergent promoters that control the *ipa* (P*icsB*) and *mxi* (P*ipgD*) operons have been more thoroughly characterized [34,57]. The *icsB* promoter was initially found to be H-NS-responsive using a P*icsB-lacZ* transcriptional reporter in an *E. coli* background. Subsequently, electrophoretic mobility shift assays (EMSAs) and DNase I footprints revealed that H-NS binds the *icsB-ipgD* intergenic region, protecting sequences between +60 to −220 relative to the *icsB* transcription start site (TSS) [34]. Other analyses in this study suggest that the precise step of transcription initiation to be inhibited by H-NS is open complex formation, but because binding of RNA polymerase to the promoter was not demonstrated in the presence of H-NS, it remains unclear if RNA polymerase binding itself or the formation of the transcriptional bubble is actually the step inhibited by H-NS [34]. The transcriptional anti-silencing of P*icsB* requires a VirB-binding site centered 120 bp upstream of the *icsB* TSS. Based on biochemical and structural studies [56,58,59], a counter-silencing mechanism has been proposed in which VirB binds to its site, induces a local bend, and oligomerizes along the DNA, thus destabilizing the downstream H-NS silencing complex ([56]; Figure 3B). At the divergent promoter *ipgD*, evidence for H-NS-mediated silencing comes from two different *lacZ* transcriptional reporters: an *ipgD*-*lacZ* reporter in *E. coli* [57] and a *lacZ* reporter located in the second to last gene of this fifteen gene operon in *S. flexneri* [37], as well as Northern analysis in *S. flexneri* [39]. While it remains unclear if the same sequences required for H-NS-mediated silencing and VirB-dependent anti-silencing of the *icsB* promoter are also required for the regulation of the *ipgD* promoter, this does seem likely given the close proximity of these two promoters.

An example of a VirB–regulated gene that lies outside of the *ipa-mxi-spa* region is *icsP*. Promoter activity of this gene was measured using a plasmid-borne P*icsP-lacZ* reporter in both *E. coli* and *Shigella* backgrounds [50,52]. While 5′ truncation analyses of the *icsP* promoter reveal that the DNA sequences located between 800 and 350 bp upstream of the primary *icsP* TSS are required for H-NS-mediated repression [60], the VirB binding site required for transcriptional anti-silencing of this promoter lies over 1 kb upstream of the primary *icsP* TSS [52,61]. To date, it remains unclear how silencing and anti-silencing of the *icsP* promoter occurs from such remote sites.

The final step of the virulence gene cascade is triggered by the VirB-dependent up-regulation of the *mxi* operon because *mxiE* encodes the last transcriptional regulator of this cascade (Figure 2). In response to activation of the type III secretion system, MxiE and its co-activator IpgC transcriptionally activate a suite of genes encoding type III secretion effector molecules. While some of these genes are also regulated by VirB [51], to the best of our knowledge, the regulatory interplay of MxiE and IpgC, VirB and H-NS at these promoters has not been studied.

In summary, the effect of H-NS on *Shigella* virulence gene expression is pervasive (Figure 2). A switch to 37 °C is absolutely required to alleviate H-NS-mediated silencing and is caused either by a temperature-induced change in DNA topology (Figure 3A) or through the temperature-dependent production of VirB, a counter-silencing protein (Figure 3B). Mechanistically, H-NS-mediated silencing appears to rely on the occlusion of promoter elements by H-NS (Figure 3A,B). Importantly however, with the exception of work done at the *virF* promoter [32] where an in vivo dimethyl sulfate footprint was employed, most evidence in the *Shigella* literature supporting H-NS-mediated promoter occlusion comes from in vitro DNase I protection assays [33,34,57]. Importantly, these studies do not show promoter occlusion per se, but simply show H-NS occupying promoter regions in the absence of all other proteins. Given that promoter regions are AT-rich, perhaps it is not surprising to find that H-NS binds to promoter regions in vitro. At other virulence plasmid loci, including the *icsP* region, the region required for H-NS-mediated silencing does not overlap promoter elements [60]. This raises the possibility that other H-NS binding regions have yet to be identified or that other mechanisms of H-NS-mediated silencing exist. In fact, other models of silencing have been proposed that suggest H-NS may interfere with transcription elongation rather than transcription initiation [62,63,64]. Furthermore, the recent finding that H-NS is tyrosine phosphorylated in *S. flexneri* 2457T [65], raises the possibility that post-translational modification of H-NS may regulate H-NS:DNA interactions and cause changes in H-NS-mediated silencing of virulence genes in this bacterium. Clearly, there is still much to be learned about the role of H-NS in this important human pathogen.

## 3. Activities of the H-NS Paralogues StpA and Sfh and Their Possible Roles in Virulence Gene Regulation

The two other H-NS family members found in *Shigella* spp., —StpA (58% identical to H-NS and 100% identical to StpA in *E. coli*) and Sfh (59% identical H-NS), —share a similar domain organization to H-NS [[15]; Figure 1). As a consequence, all three proteins have the ability to interact with one another to form heteromeric complexes [15]. In addition, StpA and Sfh can complement various H-NS-dependent phenotypes in *E. coli*, restoring Bgl (ability to use β-glucosides) and mucoidy phenotypes to those exhibited by wild type [66], leading to the proposal that StpA and Sfh primarily serve as a backup system for H-NS in *Shigella* [15,66,67]. In support of this, *stpA* and *sfh* transcripts are significantly elevated in an *hns* mutant background, whereas *hns* transcript levels are not significantly altered in *stpA* or *sfh* null mutants [15]. As “molecular backups” for H-NS, these proteins may mitigate potentially harmful effects caused by either the occasional loss of a functional *hns* gene or the acquisition of AT-rich DNA by horizontal gene transfer [12,67,68]. Although only two studies have examined the regulatory activities of the H-NS paralogues in *Shigella* [15,66], the activities of these proteins have been studied more extensively in *E. coli* or *Salmonella* [16,30,69,70]. Since some of these reported activities may influence virulence gene regulation in *Shigella* spp., we will review these activities before describing our current understanding of the role that StpA and Sfh play in *Shigella* virulence gene regulation.

### 3.1. Reported Activities of StpA and Sfh in E. coli and Salmonella

In *E. coli*, StpA serves to supplement the H-NS pool through its transient expression during exponential growth [6,71]. The routine presence and conserved location of *stpA* in *E. coli*, *Salmonella* and *Shigella* strains strongly suggests that this paralogue arose through an *hns* gene duplication event that predates the divergence of these bacterial clades from one another [72]. Like H-NS, StpA constrains DNA supercoils and forms bridges and nucleoprotein filaments on AT-rich DNA exhibiting planar curvature [16,30,73,74]. Interestingly, StpA filaments are more stable than H-NS filaments when measured in vitro [75], suggesting the regulatory activities of StpA and H-NS may differ. While initial identification of StpA was as a protein that suppresses a splicing defective *td*^-^ intron mutant from bacteriophage T4 when overexpressed in vivo [76], subsequent in vitro studies revealed that its RNA chaperone activity was responsible for this phenotype, promoting proper RNA folding and the formation of a competent, self-splicing intron [16,77,78]. Indeed, StpA binds RNA more strongly than H-NS, an activity that is demonstrated to promote intra-strand RNA annealing or strand displacement [79]. Another example of StpA serving as a regulator of RNA activity can be seen in *E. coli,* where StpA destabilizes the sRNA MicF. Since MicF functions to block translation of the OmpF porin, the actions of StpA make it a negative post-transcriptional regulator of MicF. Interestingly, H-NS also negatively regulates MicF levels but does so by repressing transcription and hence the production of this sRNA [80,81]. Thus, it is clear that at certain genetic loci, StpA and H-NS are not functionally redundant, but instead, serve independent and distinct roles as regulators of gene expression.

In contrast to StpA, much less is known about the Sfh protein. Sfh shares higher amino acid identity with StpA than H-NS (62% versus 59%, respectively). Unlike StpA, Sfh is encoded by the R27-like conjugative plasmid found exclusively in *S. flexneri* strain serotype 2a, 2457T [15]. While it remains unclear how or when this plasmid was acquired by 2457T, evidence from *Salmonella enterica* Typhimurium suggests that Sfh facilitated the acquisition of this AT-rich plasmid by preventing native pools of H-NS being titrated away from AT-rich sequences located on both the *Shigella* chromosome and virulence plasmid [69,70]. In support of this hypothesis, when a copy of the R27-like plasmid lacking *sfh* was introduced into *S*. *enterica* Typhimurium, the expression of over 300 genes was altered when compared to the wild type plasmid [69,70]. Doubts about this titration hypothesis, however, have been raised because many of the genes showing altered expression were not identified as H-NS-regulated in previous studies and multiple bona fide H-NS-regulated genes were not affected by the introduction of the Δ*sfh* plasmid [82]. Regardless, it does appear that the presence of Sfh has enabled 2457T to acquire the R27-like conjugative plasmid by minimizing fitness defects, suggesting more broadly that the presence of H-NS-like proteins on conjugative plasmids may facilitate their dissemination to a variety of different bacterial populations [82].

### 3.2. Important Considerations When Studying Virulence Gene Regulation by the H-NS Family

The presence of three H-NS family members in *S. flexneri* strain 2457T raises questions about their expression profiles. Protein levels of each H-NS family member found in 2457T have been measured in wild type cultures grown in LB broth at 37 °C [15,17]. While H-NS levels remain relatively constant throughout growth, StpA protein levels peak in early exponential phase and drop precipitously to undetectable levels in stationary phase cultures. In contrast, Sfh protein levels follow an opposite pattern, where they are very low in exponential growth and increase about 2.5-fold as cultures enter stationary phase. These different expression profiles support the idea that each member of the H-NS family may serve a discrete, physiological role in *Shigella.* Furthermore, they allow hypotheses to be generated about when heteromeric interactions between family members are most likely to occur (if they occur), and when *Shigella* virulence gene expression is likely regulated by individual proteins or combinations thereof, at least under the conditions tested.

**Figure 4:**
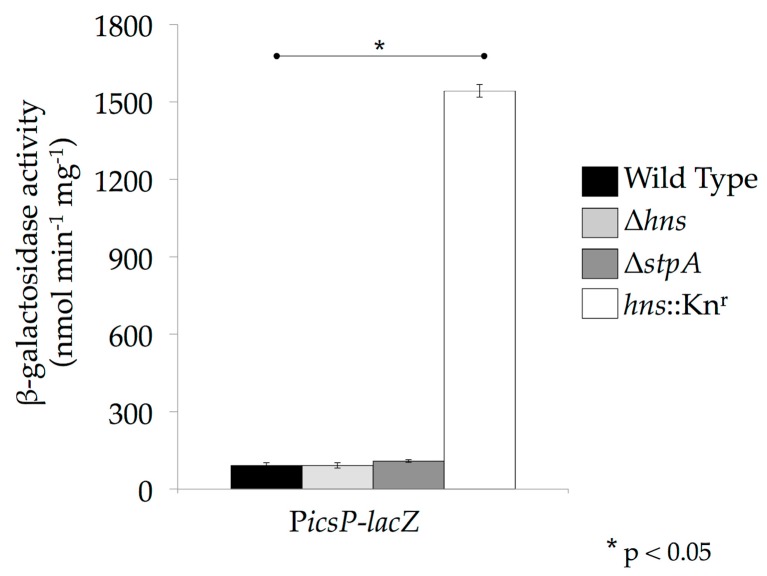
StpA and Sfh can silence the *icsP* promoter in the absence of H-NS. Activity of the P*icsP-lacZ* reporter was measured in the absence of H-NS or StpA in *E*. *coli* MC4100 and its isogenic derivatives. β-galactosidase activity was measured after cells had been grown at 37 °C, as described previously [50]. Strains denoted Δ*hns* and Δ*stpA* are true null mutants, whereas *hns:*:Kn^r^ carries a dominant-negative allele. Representative data of three independent trials are shown. A Student’s T-test was performed for statistical significance, * *p* < 0.05.

**Figure 5:**
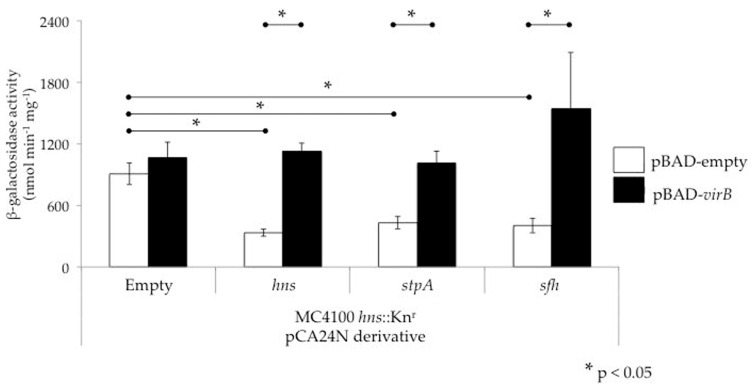
VirB can alleviate silencing mediated by H-NS family members at the *icsP* promoter. Activity of the P*icsP-lacZ* reporter was measured in an *E. coli* MC4100 *hns* dominant-negative mutant carrying two additional inducible plasmids (pCA24N or its derivatives bearing an *hns* family member and pBAD or its derivative bearing *virB*). Each *hns* family member was induced (0.2 mM IPTG) from the pCA24N series throughout growth in Luria-Bertani broth at 37 °C for 5 h. In the last hour of growth, *virB* was induced with L-arabinose (0.2% w/v). Assays were conducted in triplicate and representative data of three independent trials are shown. A Student’s T-test was performed for statistical significance, * *p* < 0.05.

Another important consideration when studying virulence gene regulation by the H-NS family in *Shigella* is the nature of the *hns* mutant being used. The *hns* mutant alleles used in *Shigella* studies (*hns*::Tn10; [36] & *hns*::Kn^r^; [33]) are not true null mutants, but rather encode heavily truncated proteins comprised of the N terminal oligomerization domain or part of it (1–93 or 1–37 amino acids, respectively) ([20,50,66,83]; Figure 1). Since H-NS, StpA and Sfh are known to oligomerize with themselves as well as with each other [15,66], these truncated derivatives of H-NS are capable of interacting with StpA and Sfh and partially disrupting their nucleic acid binding activity in a dominant-negative manner [20]. Consequently, use of dominant-negative *hns* mutant alleles is likely to mask the activities of these proteins. This is supported by data collected at the *icsP* promoter where promoter activity was measured in a wild type *E. coli* background or in isogenic derivatives containing either an *hns* dominant-negative allele or *hns* or *stpA* nulls. In the *hns* or *stpA* null backgrounds, activity of the *icsP* promoter was repressed similar to wild type, but was significantly higher in the *hns* dominant-negative mutant background (Figure 4). These findings are consistent with previous studies that have characterized these mutant alleles [84]. The data presented in Figure 4 demonstrate a clear phenotypic difference between the *hns* dominant-negative mutant and null alleles, which may explain why dominant-negative alleles rather than *hns* null mutants have been used in studies of *Shigella* virulence. Interestingly though, when *hns* or its family members were induced from pBAD plasmids in the *hns* dominant-negative background, *icsP* promoter activity decreased significantly (Figure 5; compare the white bars). These data and those presented in Figure 4 are consistent with the *hns* dominant-negative allele masking the activity of native H-NS family members, but demonstrate that any of the *hns* paralogues can overcome this dominant-negative effect when they are overexpressed. These findings clearly highlight the importance of understanding the type of *hns* mutant allele being used in an experiment so that the resulting data can be interpreted appropriately.

### 3.3. The Role of StpA and Sfh in Shigella Virulence Gene Regulation

Our understanding of the involvement of StpA and Sfh in virulence gene regulation in *Shigella* is relatively rudimentary compared to H-NS and is limited to analyses of the H-NS-silenced and VirB-anti-silenced *mxi* operon. These analyses were carried out in a 2457T derivative named BS184 [36], which contains a *mxiC::lacZ* transcriptional fusion in the large *mxi* operon located on the virulence plasmid. As expected for an H-NS-regulated locus, *lacZ* production in BS184 is low at 30 °C, but elevated at 37 °C in stationary phase cultures, consistent with the well-characterized temperature-dependent regulation of virulence genes in *Shigella* [36]. Furthermore, a BS184 derivative carrying an *hns* dominant-negative mutant allele showed elevated levels of *lacZ* activity at 30 °C [66], consistent with loss of H-NS-mediated silencing [36] and data collected at the *icsP* promoter (Figure 4). This phenotype is likely caused by both the loss of functional H-NS and the partial negative effect that the heavily truncated H-NS protein has on the activities of StpA and Sfh because BS185 carries an *hns* dominant-negative allele. In contrast, *lacZ* production in BS184 derivatives carrying clean deletions of *sfh*, *stpA* or both was similar to that observed in the BS184 wild type background at 30 °C and 37 °C. This finding strongly suggests that StpA and Sfh do not play a regulatory role at this locus in the presence of H-NS [66]. Interestingly, when the *hns* dominant-negative allele was combined with the *sfh* deletion, *lacZ* activity increased at 30 °C above levels observed in the *hns* mutant, suggesting that Sfh may participate in the regulation of the *mxi* locus, but only in the absence of functional H-NS.

In the same study, overexpression of either *stpA* or *sfh* in the BS184 background led to a decrease in *lacZ* expression at 37 °C [66]. Under these experimental conditions, VirB protein levels were dramatically decreased, most likely because overexpression of the paralogues had led to the silencing of the *virB* promoter, the *virF* promoter or both. This decrease in *ipgD* promoter activity may have been caused by the paralogues themselves or through a loss of VirB anti-silencing. Support for the indirect modulation of the *mxi* operon by StpA and Sfh was provided by EMSAs using DNA fragments taken from the *virB* and *virF* promoters. Recombinant H-NS, StpA and Sfh proteins were shown to bind to these DNA sequences in a concentration-dependent manner. It is surprising that DNA fragments taken from the *ipgD* promoter were not used in this experiment because this promoter controls the *mxiC::lacZ* transcriptional reporter in BS184. It is also unfortunate that the stability of the >9 kb *mxi* transcript generated prior to the *lacZ* fusion was not examined in the presence and absence of the paralogues because StpA, at least, has been shown to modulate RNA stability [79,80].

In summary, it is clear that StpA and Sfh can be a substitute for H-NS, but it is not known whether the silencing imparted by these proteins is mechanistically identical to that mediated by H-NS. Furthermore, it remains unclear if the backup role exhibited by StpA and Sfh in these assays is the only regulatory effect that these proteins impart in the context of *Shigella* virulence gene expression.

## 4. Can VirB Alleviate StpA- and Sfh-Mediated Silencing?

The observation that StpA and Sfh can silence virulence genes, albeit in the absence of H-NS, not only raises questions about the silencing activities of these proteins, but also about their interplay with the *Shigella* counter-silencing protein VirB. To address this, we tested whether VirB can alleviate silencing mediated by either of the two H-NS paralogues StpA and Sfh at the *icsP* promoter. To do this, an *E*. *coli hns* dominant-negative background was used to avoid some of the complications that arise when using similar mutant alleles in *Shigella,* including artifacts caused by the instability of the virulence plasmid and indirect effects caused by the up-regulation of both *virF* and *virB* genes. In the *E. coli hns* dominant-negative mutant background, overexpression of *hns*, *stpA* or *sfh* leads to silencing of the *icsP* promoter (Figure 5; compare the white bars). Moreover, expression of *virB* from an inducible promoter relieved this silencing (Figure 5; compare the black bars to the white bars). Consequently, these data show that in addition to H-NS, VirB can alleviate transcriptional silencing mediated by StpA or Sfh. While the relevance of this finding in the context of *Shigella* physiology remains unclear, evidently VirB does have the ability to relieve silencing mediated by StpA or Sfh if required to do so.

## 5. Conclusions and Perspectives

Over 25 years ago, the importance of H-NS as a regulator of *Shigella* virulence was established [36]. Despite this, questions remain about the underlying mechanisms of H-NS-mediated silencing of virulence genes. While a mechanism of promoter occlusion appears to be fairly common, conclusive experimental support has been collected at only a few genetic loci. The finding that some regions on the *Shigella* virulence plasmid do not overlap promoter elements [47] may indicate that alternative mechanisms are at play. Indeed, alternative mechanisms of H-NS-mediated silencing have been proposed, including those that result in H-NS interfering with other steps of transcription initiation [62,63,75] or the process of transcriptional elongation [85]. Consequently, it seems likely that more than one mechanism of H-NS-mediated silencing may be involved in the regulation of *Shigella* virulence genes.

In this regard, if we are to more thoroughly understand H-NS-mediated silencing in *Shigella*, a global in vivo approach will be required. Indeed, the identification of sites bound by H-NS in vivo at 30 °C and 37 °C and in the presence and absence of *virB* would provide valuable insight into virulence gene regulation in *Shigella* and provide a foundation for studies that seek to clarify mechanisms of silencing and anti-silencing. On a separate but related topic, the recent finding that H-NS family members are phosphorylated in *S. flexneri* [65] and *E. coli* [86] raises the possibility that post-translational modification of H-NS family members may also regulate interactions with DNA, triggering changes in the silencing of virulence genes. Clearly, it is an exciting time to be studying the H-NS family of proteins and their role in virulence gene regulation.

The observation that *Shigella* spp. contain one and sometimes two H-NS paralogues adds another layer of complexity to virulence gene regulation in this pathogen. Most of the evidence gathered in *Shigella* suggests that the paralogues act as a functional substitute for H-NS, a finding that is further supported by VirB alleviating either StpA- or Sfh-mediated silencing of the *icsP* promoter (Figure 5). In contrast, other lines of evidence support the possibility that the paralogues exhibit additional regulatory roles. These include the observations that StpA can regulate RNA stability [79,80], the in vitro properties of StpA and H-NS filaments differ from one another [75] and all three H-NS family members display distinct expression profiles in *S. flexneri* strain 2457T [15]. It is possible that some of these additional regulatory activities have been obscured by the exclusive use of *hns* mutants bearing dominant-negative alleles in *Shigella* research. Nevertheless, through careful experimentation, additional roles of StpA and Sfh and their regulatory effects on virulence gene expression may be identified in *Shigella*.

In order to get a clear view of the role of each H-NS family member, it will be important to study each protein in the absence of the other family members. The use of null mutants, alone or combination, may elucidate how these proteins function independently and coordinately to regulate virulence genes. The risk of these studies is that the remaining protein may or may not be present at levels found in wild type cells due to the fact that H-NS family members are capable of negative auto- and cross-regulation [15]. This would need to be investigated because elevated or diminished levels of protein could lead to phenotypic artifacts. An alternative approach would be to delete all genes encoding the family members and to reintroduce each gene independently in trans. The downside of this approach is that, at least in *E. coli,* mutants lacking all genes encoding H-NS family members are prohibitively sick [87,88]. Nevertheless, with careful planning and the use of multiple approaches to validate observed activities, it seems likely that a clearer understanding of the roles exhibited by the H-NS paralogues found in *Shigella* will be achieved.

A further layer of complexity arises from the finding that all three H-NS family members are capable of forming heteromers with each other, at least in a yeast two-hybrid assay [15]. Currently, evidence to support the natural formation of H-NS family heteromers in *Shigella* is lacking. The tendency of these proteins to form heteromers, however, is an important consideration for investigators because heteromeric complexes may form when two or more H-NS family members are expressed together at high levels. Furthermore, it is possible that the resulting heteromers will have different activities than their homomeric counterparts.

To conclude, *Shigella* presents a fascinating opportunity to study the relationship between H-NS, StpA and Sfh and their role in the regulation of virulence gene expression in this important human pathogen. The presence of two or more of these nucleoid structuring proteins in all *Shigella* species, pathogenic *E. coli* and *Salmonella* strains and many other bacterial pathogens elevates interest in this family of proteins that are intricately tied to bacterial physiology and virulence. Since loss of H-NS family members causes severe growth defects in some bacteria, H-NS and its paralogues may constitute prime molecular targets for novel antibacterial therapies.
