# Erratum: Michael A. Picker; et al.; H-NS, Its Family Members and Their Regulation of Virulence Genes in *Shigella* Species. *Genes* 2016, *7*, 112

**DOI:** 10.3390/genes8060162

**Published:** 2017-06-15

**Authors:** Michael A. Picker, Helen J. Wing

**Affiliations:** School of Life Sciences, University of Nevada Las Vegas, Las Vegas, NV 89154-4004, USA; pickerm3@unlv.nevada.edu

The authors wish to make the following change to their paper [1]. In the Acknowledgments Section, the sentence “The IPTG-inducible pCA24N (-*gfp*) plasmid and derivatives were used to express each of the *hns* family members found in *Shigella flexneri* 2a strain 2457T. We acknowledge National BioResource Project: *E. coli* at NIG for sending us these ASKA clones [89].” should be inserted after “We thank……editorial comments”. The added reference [89] is “89. Kitagawa, M.; Ara, T.; Arifuzzaman, M.; Ioka-Nakamichi, T.; Inamoto, E.; Toyonaga, H.; Mori, H. Complete set of ORF clones of *Escherichia coli* ASKA library (a complete set of *E. coli* K-12 ORF archive): Unique resources for biological research. *DNA Res.* **2005**, *12*, 291–299.”

We apologize for any inconvenience caused to the readers by this omission. The manuscript will be updated and the original will remain online on the article webpage.
